# Pseudomonas Reinfection in a Patient With Primary Ciliary Dyskinesia: How a Toothbrush Could Make You Sick

**DOI:** 10.7759/cureus.83047

**Published:** 2025-04-26

**Authors:** Jose Colon-Soto, Arnaldo J Santos-López, Francisco Alvarado-Huerta, Jesus M Melendez Montañez, Wilfredo De Jesús-Rojas

**Affiliations:** 1 Pediatrics, Ponce Health Sciences University, Ponce, PRI; 2 Pediatrics, Saint Luke’s Hospital, Ponce, PRI; 3 Pediatrics, University of Puerto Rico, Medical Sciences Campus, School of Medicine, San Juan, PRI; 4 Pediatrics, University of Pittsburgh Medical Center, Children's Hospital of Pittsburgh, Pittsburgh, USA

**Keywords:** built environment, infection, primary ciliary dyskinesia, pseudomonas aeruginosa, toothbrush

## Abstract

Primary ciliary dyskinesia (PCD) is a rare autosomal recessive disorder characterized by impaired mucociliary clearance, leading to recurrent respiratory tract infections, chronic rhinosinusitis, and progressive bronchiectasis. *Pseudomonas aeruginosa* is a common pathogen associated with poorer clinical outcomes in patients with PCD. While environmental sources of *P. aeruginosa* are well-documented in cystic fibrosis (CF) patients, to our knowledge, this is the first reported case implicating toothbrush colonization as a likely source of recurrent *P. aeruginosa* infections in a patient with PCD. We present a case of a 53-year-old Puerto Rican male with a history of sinusitis, bronchiectasis, hearing loss, and chronic cough, diagnosed with PCD, having a positive genetic testing for *RSPH4A* (c.921+3_921+6del (intronic)) founder mutation. Despite aggressive treatment, the patient continued to experience recurrent *P. aeruginosa* infections. Investigation into potential environmental sources revealed that the patient's toothbrush was colonized with *P. aeruginosa*, making it a likely source of reinfection. After modifying his oral hygiene practices, the patient showed significant clinical improvement with no subsequent hospitalizations. This case highlights the novel identification of a toothbrush as a source of recurrent *P. aeruginosa* infections in a patient with PCD. It underscores the importance of considering environmental factors in the management of chronic respiratory infections in patients with PCD. These findings suggest that routine evaluation and disinfection of hygiene tools, such as toothbrushes, may be critical in preventing recurrent infections and their long-term consequences in patients with PCD. Future research should aim to establish clinical guidelines for preventing bacterial transmission from the built environment in this vulnerable population.

## Introduction

Ciliopathies are a collection of disorders related to cilia dysfunction [1]. Primary ciliary dyskinesia (PCD) is a rare autosomal recessive ciliopathy marked by various clinical manifestations, such as persistent or recurrent infections in the upper and lower respiratory tracts, defects in laterality (including situs inversus totalis and heterotaxy), and infertility in males. [2]. Diagnosing PCD is complex due to its genetic diversity, varied clinical manifestations, and the limited availability of specialized diagnostic tools [3]. According to the American Thoracic Society (ATS) Clinical Practice Guideline, the diagnosis of PCD requires a combination of characteristic clinical features along with supportive diagnostic testing such as low nasal nitric oxide levels, high-speed video microscopy, transmission electron microscopy, and/or identification of biallelic pathogenic variants in a known PCD-associated gene [4]. Patients with PCD exhibit impaired mucociliary clearance, predisposing them to recurrent infections of the upper and lower respiratory tracts, chronic rhinosinusitis, progressive bronchiectasis, and atelectasis [5]. The most isolated pathogen from patients with PCD is Haemophilus influenzae [6,7]. Other common pathogens were *Pseudomonas aeruginosa*, *Streptococcus pneumoniae*, *Moraxella catarrhalis*, and *Staphylococcus aureus* [6,7]. Patients with PCD often present with bronchiectasis, for which the presence of *P. aeruginosa* has been associated with poorer clinical outcomes [8]. Cases have been reported where colonization with *P. aeruginosa* affects lung structure and function in patients with PCD over the long term, leading to post-infectious bronchiolitis obliterans (PIBO) [9].

Despite the different pathophysiology, chronic infections and their long-term consequences in patients with PCD often overlap with those seen in cystic fibrosis (CF) [6]. *P. aeruginosa* is one of the most commonly isolated pathogens in the airways of patients with CF [10]. Most patients with CF develop chronic *P. aeruginosa* infections by their teenage years, or even earlier, and these respiratory infections contribute significantly to the morbidity and mortality associated with the disease [11]. There have been reports of cases of patients with CF with recurrent/chronic respiratory infections linked to the presence of biofilms and live pathogens in toothbrushes [12-14]. On the other hand, to our knowledge, there have not been any published reports of cases of PCD patients with recurrent/chronic respiratory infections linked to the presence of alive pathogens in toothbrushes. In this case report, we present the first adult case of a recurrent *P. aeruginosa* respiratory tract infection related to toothbrush colonization in a Puerto Rican patient with PCD with the founder genetic mutation.

## Case presentation

This is a case of a 53-year-old Puerto Rican male patient with a past medical history significant for sinusitis, bronchiectasis, hearing loss, and chronic cough that presented for evaluation due to recurrent sinopulmonary *P. aeruginosa* infections. The patient has a negative family history of reported genetic abnormalities associated with respiratory defects; however, no family members were available for genetic testing to confirm carrier status. He has experienced worsening pulmonary infections over the past 10 years, leading to six hospitalizations and intravenous antibiotic home therapy. Initial treatment included cefepime and levofloxacin, which over time the patient developed resistance. After seeking a pulmonary specialist, the patient was initially diagnosed with asthma. Later on, the patient was referred to an otorhinolaryngologist (ENT) for evaluation of possible sinusitis. The patient was diagnosed with chronic sinusitis, and functional endoscopic sinus surgery was performed. After surgery, his sinusitis worsened, and he was referred to the Puerto Rico PCD Center for diagnostic evaluation due to suspected PCD.

**Figure 1:**
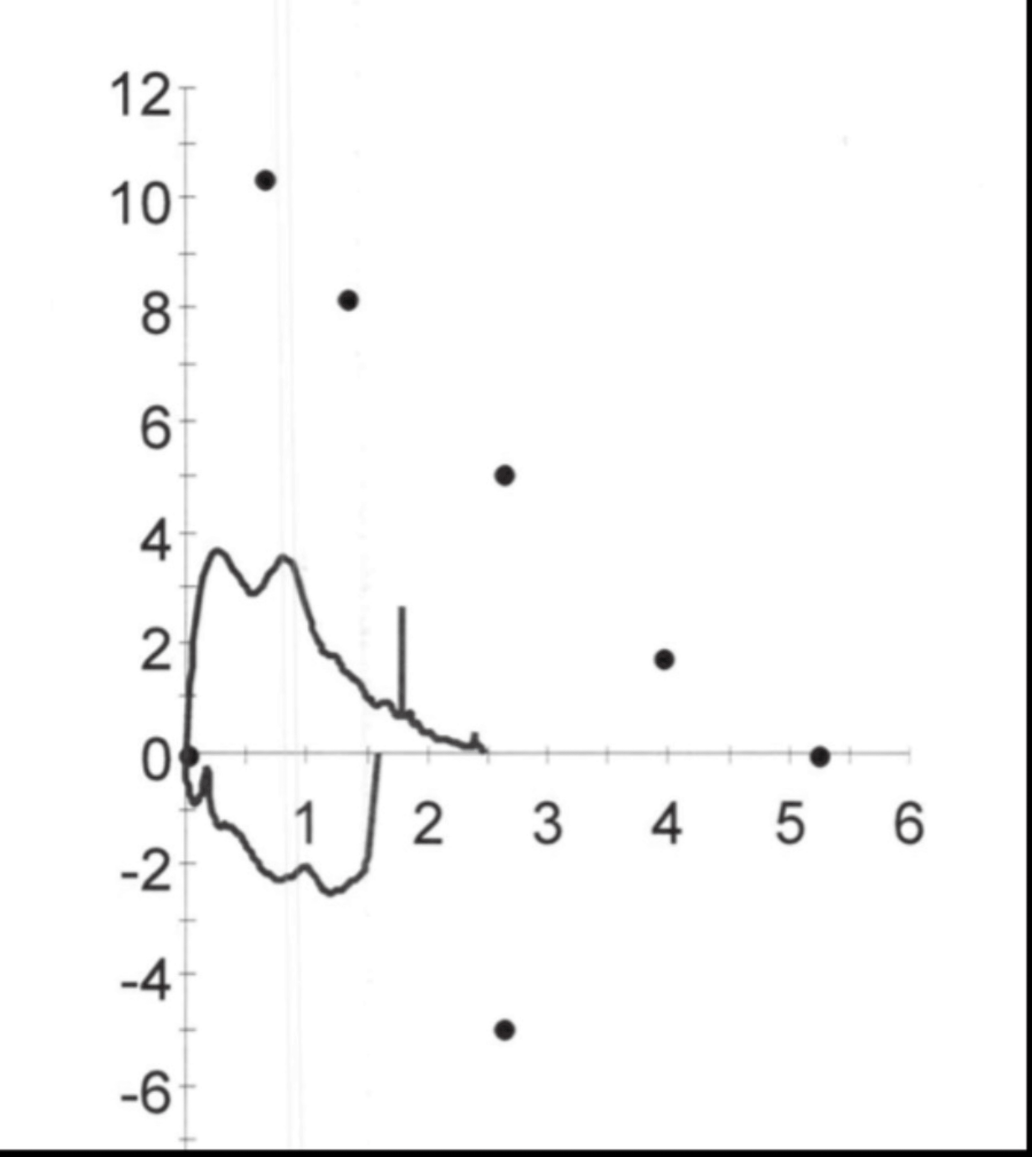
Flow-Volume Loop: Patient’s flow-volume loop consistent with a restrictive process, as seen by the diminished size of the loop.

**Figure 2:**
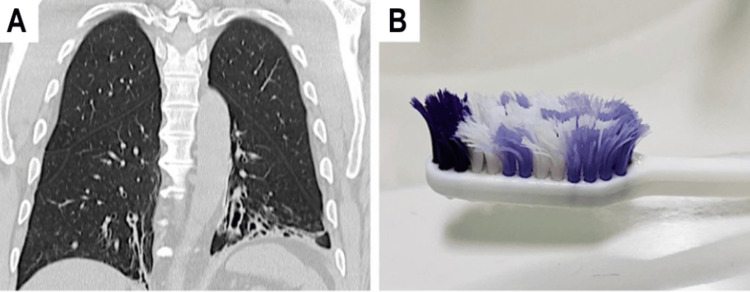
Chronic reinfection in a patient with primary ciliary dyskinesia (PCD) due to colonized toothbrush with Pseudomonas aeruginosa. (A): Coronal view of a chest CT scan showing bibasilar bronchiectasis in a 53-year-old male with PCD homozygous for the RSPH4A (c.921+3_921+6del (intronic)) founder mutation. This patient with PCD is colonized with mucoid *P. aeruginosa*, and regardless of aggressive therapeutic approaches patient continued with recurrent sinopulmonary exacerbations. (B)The toothbrush of the same patient with PCD was used in the past two months as part of his oral hygiene routine. Bacterial cultures were positive for the presence of *P. aeruginosa* on the toothbrush.

Upon physical examination, the patient is noted to have an obese habitus but is otherwise active, alert, and cooperative. Lung examination reveals fair air entry bilaterally with crackles at the bases and rhonchi on auscultation. No cyanosis or clubbing was noted on the extremities. The remainder of the examination, including cardiovascular, abdominal, neurological, and skin assessments, was unremarkable, with no significant findings of clubbing. The patient was screened with the Primary Ciliary Dyskinesia Rule (PICADAR), a clinical prediction tool based on patient history and symptoms to estimate the likelihood of PCD, where he scored a total of 12 points, indicating a 90% chance of PCD [15]. Additionally, pulmonary function tests (PFTs) showed the following results - forced vital capacity (FVC): 47%, forced expiratory volume (FEV1): 43%, FEV1/FVC: 92%, and forced expiratory flow (FEF) 25-75%: 35% of percentage predicted, consistent with a restrictive airflow pattern (Figure 1). A high-resolution chest computer tomography (HRCT) scan showed bibasilar bronchiectasis and mucus plugs (Figure 2A). Furthermore, the nasal nitric oxide (nNO), measured using a chemiluminescence analyzer, averaged 5.2 nL/min, with measurements repeated twice on separate days, two weeks apart, during periods of baseline cough and symptoms. A sweat test was normal.

**Figure 3:**
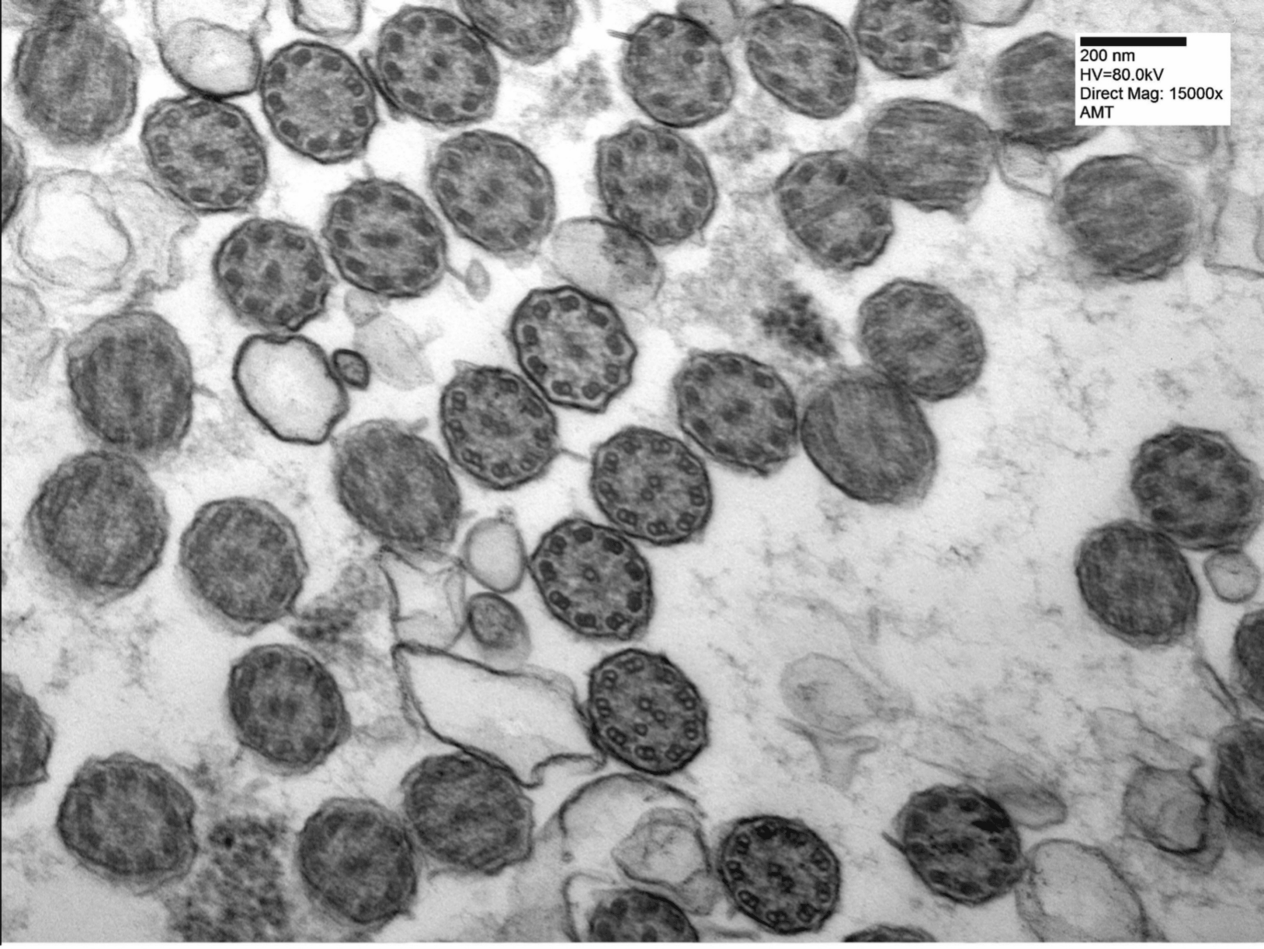
Nasal Biopsy: Patient’s nasal biopsy showed cilia with occasional ultrastructural abnormalities, including central complex defects (9 + X) and partial loss of axis orientation.

Based on the patient’s medical history, physical examination, PICADAR result, PFTs results, imaging findings consistent with bronchiectasis and low nNO, genetic testing for PCD and CF was performed. Genetic testing revealed a central complex ultrastructural defect and homozygous presence of the founder Puerto Rican genetic mutation RSPH4A (c.921+3_921+6del (intronic)) founder mutation. Additionally, a nasal biopsy was performed, which showed cilia with occasional ultrastructural abnormalities, including central complex defects (9 + X) (Figure 3) and partial loss of axis orientation, most consistent with PCD. In this case, the diagnosis of PCD was supported by a combination of clinical features, a high PICADAR score, markedly low nNO, genetic confirmation of the RSPH4A c.921+3_921+6del founder mutation, and nasal biopsy findings demonstrating occasional ultrastructural abnormalities consistent with central complex defects. Although the ATS guidelines recommend a multimodal approach to PCD diagnosis, the presence of a pathogenic founder mutation along with supportive nasal biopsy and clinical findings provided sufficient confirmation in this patient without the need for high-speed video microscopy or repeated testing modalities [3,4].

Despite aggressive treatment with cefepime and levofloxacin, the patient had ongoing colonization with mucoid *P. aeruginosa* and sinopulmonary exacerbations. As a result, the investigation into the source of his recurrent *P. aeruginosa* infections was initiated. Upon questioning, the patient denied exposure to bathtubs, swimming, hot tubs, humidifiers, or swimming in lakes or rivers, which are commonly associated with *P. aeruginosa* infection. Upon inquiry about his oral hygiene and daily routines, the patient revealed that he followed a consistent hygiene regimen but had not replaced his toothbrush (Figure 2B) or shower head in over a year. He was asked to bring in his shower head, metal shower tube, and toothbrush for evaluation. Bacterial cultures were performed on the patient’s sputum and toothbrush, and only the toothbrush yielded positive results for the presence of *P. aeruginosa*. Non-speciated Gram-positive bacteria were also detected on the shower head and metal shower tube. Given these findings, the toothbrush is considered a possible source of recurrent infection in this patient.

Treatment and outcome

The patient received a comprehensive airway clearance regimen, which included bronchodilators, hypertonic saline 7%, and high-frequency chest wall oscillation (HFCWO). Due to the presence of bronchiectasis, chronic azithromycin therapy (250 mg orally, three times per week) was initiated to reduce the frequency of exacerbations through its anti-inflammatory effects. To mitigate potential sources of recurrent infection, the patient was advised to modify his hygiene practices, including replacing his toothbrush every three months and changing his shower head every six months. Following these interventions, the patient experienced a significant improvement, with no hospitalizations due to bronchiectasis exacerbations during a two-year follow-up period. Repeated cultures still growing *P. aeruginosa* in sputum were attributed to chronic airway colonization, a common finding in patients with PCD due to impaired mucociliary clearance.

## Discussion

The identification of a daily-use hygiene device like a toothbrush as the source of recurrent *P. aeruginosa* infections in a patient with PCD represents a novel and significant finding that merits consideration within the PCD population. While the role of environmental sources in the transmission of *P. aeruginosa* is well-documented in patients with CF, to our knowledge, this is the first published case implicating toothbrush colonization as a direct source of reinfection in a patient with PCD. This observation is critical, as it suggests that a contaminated toothbrush can act as a persistent reservoir for pathogens, directly contributing to respiratory infections in PCD patients. Given the increased risk of chronic respiratory infections due to impaired mucociliary clearance, environmental factors such as hygiene tools must be carefully managed to minimize reinfection risk. Table 1 provides a list of potential environmental sources, but our findings indicate that toothbrushes may present an especially high risk due to repeated oral exposure, ideal moisture conditions, and frequent contamination.

**Table 1:** Potential Environmental Sources Contributing to Pseudomonas aeruginosa colonization in patients with primary ciliary dyskinesia (PCD). This table outlines common environmental sources within the built environment that may harbor *P. aeruginosa*, increasing the risk of colonization and reinfection in patients with primary ciliary dyskinesia (PCD). Table Credits: De Jesus Rojas W, Colon-Soto J

Environmental Source	Colonization Risk to the Patient
Toothbrush	Frequent exposure to moisture and oral bacteria makes toothbrushes an ideal environment for *Pseudomonas aeruginosa*, increasing the risk of reinfection.
Showerhead	Warm, moist conditions in showerheads encourage biofilm formation, allowing bacteria to thrive and potentially be inhaled during use.
Metal Shower Tube	The inner surfaces of metal shower tubes can support bacterial growth, creating conditions favorable for the colonization of pathogens like *P. aeruginosa*.
Sinks and Faucets	Constant exposure to water in sinks and faucets provides an ideal environment for biofilm and bacterial growth, increasing the risk of contamination.
Humidifiers	Humidifiers can disperse bacteria-laden mist into the air, which can then be inhaled, particularly in environments conducive to bacterial growth.
Air Conditioning Systems	Air conditioning systems trap moisture and organic material, providing a breeding ground for bacteria, which can be circulated throughout indoor spaces.
Medical Equipment	Inadequately sanitized medical equipment can harbor bacteria on surfaces, leading to reinfection during subsequent use.

Several studies have documented cases of bacterial contamination and infection related to toothbrush use in patients with CF (Table 2). One study found that the pathogens identified on toothbrushes from children, both before and during antibiotic treatment, were consistent with the species found in their sputum samples. Remarkably, *P. aeruginosa* and *S. aureus* were still able to be cultured from these children’s toothbrushes despite ongoing antibiotic treatment [12]. Another study found that *S. aureus* was present on 22% of toothbrushes used by patients and on 13% of those used by healthy children. Similarly, *P. aeruginosa* was detected on 15% of the patients’ toothbrushes and on 0-13% of the toothbrushes of healthy children. However, the effect of these findings on pulmonary colonization remains unclear [13]. Additionally, in a study involving 38 patients, including 13 adults and 25 pediatric cases, samples were collected from saliva, sputum, and used toothbrushes. Among these, at least one of the investigated species - *S. aureus*, *P. aeruginosa*, *Stenotrophomonas maltophilia*, *Achromobacter xylosoxidans*, or *Serratia marcescens* - was isolated from 60 saliva samples and 23 toothbrushes, highlighting the persistent contamination of oral hygiene tools in this population [14].

**Table 2:** Documented cases of bacterial infections in patients with cystic fibrosis linked to toothbrush use. Summary of documented cases of bacterial infections linked to toothbrush use in cystic fibrosis patients, detailing demographics, clinical presentations, and management.

Author(s) and Year	Patient Demographics (Age in years)	Clinical Presentation	Bacteria	Management
Hu et al., 2022 [12]	8-15	Recurrent lung infections	*Staphylococcus aureus*, *Pseudomonas aeruginosa*	Antibiotics
Genevois et al., 2015 [13]	8-18	Bacterial colonization of toothbrushes	*S. aureus*, *P. aeruginosa*	Not specified
Passarelli Mantovani et al., 2019 [14]	7-27	Bacterial conveyance to lower airways	*S. aureus*, *P. aeruginosa*, *Stenotrophomonas maltophilia*, *Achromobacter xylosoxidans*, *Serratia marcescens*	Not specified
Millar et al., 2020 [16]	Unknown	Bacterial colonization of toothbrushes	*S. aureus*, *P. aeruginosa*, *Burkholderia cenocepacia*	Steam disinfection

In contrast, this case demonstrates that toothbrush colonization in a PCD patient could be a likely source of reinfection. This finding is significant given that PCD patients, like CF patients, frequently develop bronchiectasis - a condition where *P. aeruginosa* colonization is linked to poorer clinical outcomes [8]. While the pathophysiology differs, the chronic infections in PCD often mirror those seen in CF [6], where *P. aeruginosa* colonization can severely impact lung function and structure, contributing to worsening bronchiectasis. Given the potential role of toothbrushes as a persistent reservoir of P. aeruginosa, our case underscores the need to re-evaluate the infection control measures in PCD management.

Toothbrush hygiene should thus be considered a crucial component of care for PCD patients. Routine evaluations of personal hygiene items, particularly toothbrushes, could be instrumental in identifying and managing chronic infection sources. Educating patients and caregivers about the importance of regularly disinfecting or replacing toothbrushes may substantially reduce the risk of bacterial transmission and subsequent respiratory infections. Moreover, another study demonstrated that steam disinfection effectively eradicated all tested organisms, including *P. aeruginosa* and *S. aureus*, from contaminated toothbrushes [16]. This suggests that steam disinfection could be a straightforward, cost-effective approach to managing bacterial contamination in PCD patients, especially as our findings indicate that toothbrushes may be underrecognized sources of infection.

Future research should focus on larger studies to investigate the prevalence of toothbrush colonization in patients with PCD and assess the efficacy of different decontamination methods. Additionally, studies comparing the genetic similarity between *P. aeruginosa* strains found on hygiene tools and those in the respiratory tract could help clarify whether toothbrushes serve as true reservoirs for reinfection. Such investigations could yield valuable data to inform guidelines aimed at preventing similar infections. Exploring the potential benefits of steam disinfection, as seen in CF patients, could also guide new strategies for managing bacterial contamination risks in PCD patients' hygiene practices.

## Conclusions

In conclusion, the identification of a toothbrush as a source of recurrent *P. aeruginosa* infections in a patient with PCD highlights the critical importance of considering environmental factors in the management of chronic respiratory infections. This case, to our knowledge, the first of its kind to implicate toothbrush colonization in a patient with PCD, underscores the need for heightened vigilance in personal hygiene practices for these patients. The overlap in the long-term consequences of chronic infections between patients with PCD and those with CF further emphasizes the potential benefits of integrating routine evaluation and disinfection of hygiene tools, such as toothbrushes, into patient care. Future research should focus on establishing clear guidelines for preventing bacterial transmission in patients with PCD, including exploring the efficacy of methods like steam disinfection that have shown promise in patients with CF.
